# Effectiveness of a family intervention on health-related quality of life–a healthy generation, a controlled pilot trial

**DOI:** 10.1186/s12889-020-08895-z

**Published:** 2020-05-29

**Authors:** Susanne Andermo, Mai-Lis Hellénius, Matthias Lidin, Ulrika Hedby, Anja Nordenfelt, Gisela Nyberg

**Affiliations:** 1 Department of Global Public Health, Karolinska Institutet, Stockholm, Sweden; 2 Department of Neurobiology, Care Sciences and Society, Karolinska Institutet, Stockholm, Sweden; 3 Department of Medicine, Karolinska Institutet, Stockholm, Sweden; 4 Theme Heart and Vessels, Karolinska University Hospital, Stockholm, Sweden; 5 The Foundation A Healthy Generation, Stockholm, Sweden; 6 The Swedish School of Sport and Health Sciences, Stockholm, Sweden

**Keywords:** Family intervention, Children, Health related quality of life and physical activity

## Abstract

**Background:**

Physical activity is associated with better health, but knowledge about health promoting interventions, including physical activity for families in disadvantaged areas and the impact on health-related quality of life (HRQOL) is sparse. The aim of this study was to assess HRQOL in children and their parents after participation in the programme “A Healthy Generation”.

**Methods:**

The programme is delivered in socioeconomically disadvantaged areas in Sweden and offers physical activity and a healthy meal or fruit twice a week from August to May to families with children in grade 2. Children (*n* = 67), aged 8–9 years, and their parents (*n* = 90) participated in this controlled study conducted in four schools, two control and two intervention schools. HRQOL of children and adults was assessed at baseline and follow-up after the intervention with the Pediatric Quality of Life Inventory (PedsQL) 4.0 and the Gothenburg Quality of Life scale, respectively. Analyses of covariance (ANCOVAs), linear regression and Pearson’s correlation were conducted.

**Results:**

There were no significant differences between intervention and control in HRQOL among children or adults after the intervention. However, in a subgroup of children (*n* = 20) and adults (*n* = 29) with initial low HRQOL scores at baseline, there was a significant difference between the intervention group and control group after the intervention (children (total score): *p* = 0.02; adults (social domain) *p* = 0.04). Furthermore, within the intervention group, there was a significant relationship between level of participation in “A Healthy Generation” and the physical domain of HRQOL among girls (*r* = 0.44, *p* = 0.01), but not boys (*r* = − 0.07, *p* = 0.58).

**Conclusion:**

Participation in the programme “A Healthy Generation” did not show a significant intervention effect on HRQOL in general. However, the findings suggest that HRQOL may be increased for children and adults with low HRQOL in disadvantaged areas. This knowledge can contribute to the development of health promoting interventions in such areas, and to more equitable health.

**Trial registration:**

ISRCTN ISRCTN11660938. Retrospectively registered 23 September 2019.

## Background

Among both children and adults, Quality of Life (QOL) or Health Related Quality of Life (HRQOL) have increasingly been used as health outcome measures [1]. There are various definitions of these concepts in the literature. The World Health Organization defines quality of life (QOL) as “an individual’s perception of their position in life in the context of the cultural and value systems in which they live and in relation to their goals, expectations, standards and concerns” [2]. HRQOL denotes aspects of the quality of life that are related to an individual’s health. It is a multidimensional concept, that involves physical, mental and social components of well-being and functioning as perceived by a person or patient or other observers [3, 4]. HRQOL is often self-reported in questionnaires. In health promoting interventions, assessment of HRQOL can guide the development of effective intervention strategies [1].

Young people generally report good health, but health is not evenly distributed. Children report better health than adolescents, and boys generally report higher HRQOL, more positive mental health and fewer psychosomatic problems than girls [5–8]. Moreover, there are socioeconomic differences in health in both children and adults [9, 10]. Previous research has shown that children in families with low socioeconomic position, as well as those with migration background, have lower self-reported health [5, 6]. Poor living standard has also been shown to be associated with increased mental health problems among children [5, 6]. In Sweden, the proportion of 13- and 15-year-old children reporting psychosomatic health problems has doubled since 1985 [6, 11]. Today, more than half of 15-year-old girls and about a third of boys the same age report multiple health complaints [6, 11]. In view of the decline in HRQOL with age, the differences with regard to socioeconomic position and gender, and the recent reports of increased psychosomatic health complaints among adolescents in Sweden, there is an urgent need to find strategies to promote health – including HRQOL – in young people. Increased physical activity (PA) has been recognized as a promising option for increasing HRQOL [12].

Regular physical activity has been associated with psychosocial, physical and cognitive health benefits in both children and adults [13–17]. Previous cross-sectional studies among children have shown that physical activity and, specifically, the amount of moderate-to-vigorous physical activity are positively associated with HRQOL and mental health [18, 19]. The social context of physical activity also matters: for example, involvement in team sports and sports at school has been shown to significantly predict higher self-reported mental health [18, 20–22]. The World Health Organization has developed physical activity recommendations for children, constituting of 60 min of daily moderate-to-vigorous physical activity [23]. However, a recent report [24] on a cross-section of Swedish children revealed that few children and adolescents reach recommended levels of physical activity and that physical activity declines during adolescence. In grade five only 29% of girls and 50% of boys reach recommended level of physical activity [24]. An inactive lifestyle and sedentary behaviour – in particular screen time among adolescents – are associated with poor mental health [25, 26]. Previous research has stressed the importance of promoting physical activity during childhood, since an active lifestyle, including participation in sport early in life, is associated with higher HRQOL, lower incidence of mental health disorders, and a greater likelihood of continuing to be active later in life [26–28]. A growing body of research has focused on physical activity interventions in the school context to promote physical activity [29], although the effect of such interventions on HRQOL is unclear. Several studies have therefore recently highlighted the importance of conducting physical activity interventions that include a broader spectrum of children’s environment to promote physical activity and HRQOL [30–33].

Patterns of physical activity among youth are influenced by individual, contextual, social and cultural factors such as families, the local community and the physical environment [31]. Parents play an important role in facilitating their children’s PA, for example through support, co-participation and encouragement [34]. Therefore, interventions aimed at the family as a whole may be a useful way to promote PA. However, little is known about the effect on such interventions on HRQOL. Additionally, most studies evaluating the effect of physical activity intervention on HRQOL in children have focused on specific groups and there is a scarcity of studies examining the effect within families in general populations. We hypothesised that there would be an increase in HRQOL after participation in the programme *A Healthy Generation* and a stronger effect in participants with initial low values of HRQOL. In addition, we expected to see a family correlation in HRQOL, since family correlations have been reported in other health-related outcomes [35, 36].

The overall aim of the current study was to assess HRQOL in children and their parents after participation in the family programme *A Healthy Generation.* Specific aims were also to evaluate whether the intervention had an effect on a subpopulation with low baseline HRQOL scores, to explore HRQOL in relation to participation, and to evaluate within-family correlations of HRQOL.

## Methods

### Design

The study was designed as a non-randomised controlled pilot trial with an intervention and waitlist control group. At time of the evaluation, the programme *A Healthy Generation* had already been implemented and is currently being delivered in 10 municipalities in Sweden in collaboration with a non-profit organisation and the local municipalities where the programmes were located. The researchers were not involved in the delivery of the programme.

### A healthy generation

*A Healthy Generation* was established in 2011 as a non-profit, politically and religiously unaffiliated foundation, with the aim to increase physical activity and encourage a healthy lifestyle among families with children aged 8–12 years. *A Healthy Generation* works in close collaboration with municipalities, local enterprises and sport associations to ensure long-term implementation of the programme. At the municipality level, a three-year contract/agreement is established between the foundation and the municipalities, and at least one health coordinator is employed at the municipality to operate the implementation of a programme developed by the foundation. The programme is implemented in schools in socioeconomically disadvantaged areas, selected by local municipalities. Children in grade 2 (8–9 years) and their families, including siblings, are invited to participate in *A Healthy Generation*. The programme *A Healthy Generation* runs over one school year, from August/September to May. It includes four intervention components, described below: 1) activity sessions offered twice a week; 2) healthy meals, either a fruit or a hot meal; 3) health information; and 4) parental support groups.

#### Activity sessions

A central aspect of the programme *A Healthy Generation* is to create opportunities for families to be physically active together as a mean to inspire further activity and create new physical activity habits. During the intervention, a total of 65 activity sessions consisting of 25–30 different types of activities were offered. Each activity lasted approximately 1 h. Examples of activities were basketball, football, dance and martial arts. The activities took place in the school’s sports hall or in other locations close to the school and were led by a health coordinator from the foundation *A Healthy Generation* together with leaders from local sports clubs (www.enfriskgeneration.se).

#### Health promoting meals/health information

Each activity session was followed by a healthy meal or fruit each week for the participating families. On weekdays, a hot meal was served in the school facilities, and on weekends fruits were offered. Health coordinators initiated informal discussions about healthy living habits during both activity sessions and at the shared meals.

#### Parental support groups

Parental support groups with external coaches were offered four times to parents, while the children participated as usual in an activity session with the health coordinators. The involvement of parents in all activities is intended to strengthen parents’ role modelling and promote a positive family environment among participating families and in relation to the local community.

### Setting and participants

**Fig. 1:**
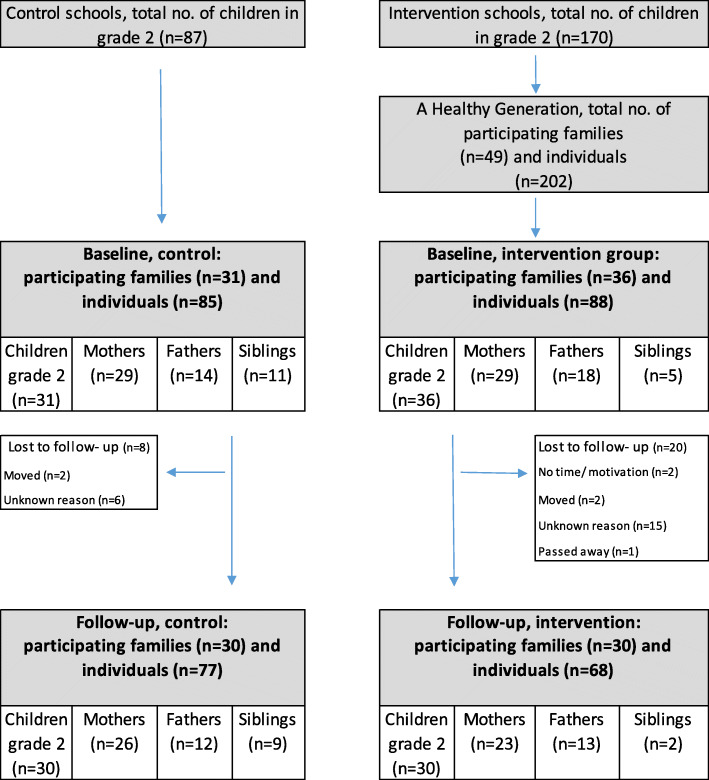
Flow chart of participants

One municipality in Stockholm, Sweden, where the programme had been in operation since 2013, was selected as a study site. This particular municipality was selected based on its location in the capital city and near the research institute. All schools (*n* = 2) in the municipality where the program was delivered were invited to participate in the study, and comparable schools (*n* = 2) in the same municipality were invited to participate as a waitlist control group. Control schools were selected based both on socioeconomic factors and for strategic reasons within the municipality, i.e. where the programme was planned to be implemented during the following year. Principals in the selected schools were invited to participate and were informed about the study. All invited schools (*n* = 4) agreed to participate in the study. A flowchart showing recruitment and retention is presented in Fig. 1. Recruitment to the study was performed by health coordinators and research assistants. Families with children attending grade two in intervention schools that had agreed to participate in the programme for the school year August 2016 – May 2017 as well as families with children in grade two in control schools were invited to participate in the study. This age group of children was selected with the intention to promote physical activity before the general decline in physical activity during adolescence [37]. Inclusion criteria were: (1) families with children in grade two in the selected schools, (2) accepted participation in the programme *A Healthy Generation* (criterion for the intervention group only).

### Data collection

Data for this study were collected at baseline (August 2016) before the intervention and at follow-up (May 2017) after the intervention. Participants were invited to their local schools to complete questionnaires and participate in physical health assessments conducted by research personnel. Participants completed questionnaires by themselves, but children were offered help when needed.

### Measures

The Pediatric Quality of Life Inventory 4.0 (PedsQL) for children aged 8–12 years, was used to measure health-related quality of life (HRQOL) in children [38, 39]. PedsQL has been translated into Swedish [40]. It is a reliable and validated instrument with an internal consistency reliability of Cronbach’s alpha = 0.88 for the total score [38, 39]. The instrument has 23 items and measures HRQOL in four domains: physical functioning (8 items), emotional functioning (5 items), school functioning (5 items) and social functioning (5 items). All items in the instrument are scored on a five-point Likert scale from 0 to 4 (0 = never, 1 = almost never; 2 = sometimes; 3 = almost always; 4 = always). The items were reversed scored and transformed to a 0–100 scale (0 = 100, 1 = 75, 2 = 50, 3 = 25, 4 = 0), with high scores indicating better HRQOL. A mean was computed by adding the sum of the items divided by the number of items. The calculation included a score for total HRQOL, a score for physical health and a score for psychosocial health: consisting of the mean of the items answered for emotional, social and school functioning.

The Gothenburg Quality of Life Instrument was used to assess QOL among adult participants in *A Healthy Generation*. The original instrument has two parts: one part for the assessment of subjective well-being and one part for the assessment of various symptoms. In this study, the 16-item well-being scale was used to measure total QOL and three domains: social, mental and physical. All items are scored on a seven-point Likert scale [1–7] with a higher score indicating a better QOL. The total score and the scores for the three domains were computed based on the sum of all questions divided by the number of answered questions in the scale and the domains. For simplicity, the term HRQOL is used when referring both to the QOL of parents and the HRQOL of children.

#### Socioeconomic position

Data on socioeconomic position were collected based on parental education through self-report. Parents were asked to state their educational level (primary, secondary, or college/university education). The answers were dichotomised so that education ≤12 years corresponded to low education, while > 12 years corresponded to high education. In the study the highest level of education achieved by either of the parents was used as an indicator of socioeconomic position for their children.

#### Country of birth

Data on parents’ country of birth were collected and dichotomised as born in Sweden or not.

#### Participation in the intervention

Data on participation in the activity sessions were collected through documentation filled in at the activity sites by the health coordinators.

### Data analysis

Data were analysed using IBM SPSS statistics version 25. Normality and heterogeneity of variance were tested for parametric assumptions. To examine potential statistically significant baseline differences in participant characteristics between groups (intervention compared to control), sub-groups (total sample compared to those with initial low HRQOL scores) and total sample vs. non-participation (< 10 times), independent t test was used for continuous variables and dichotomous variables were assessed with Chi square test.

Between-group effects (intervention/ control) were assessed by conducting analyses of covariance (ANCOVAs) on follow-up scores adjusted for baseline scores. Separate ANCOVAs were run for each outcome (total scores and sub-domains) for PedsQL and Gothenburg QOL instrument. A complete cases analysis was conducted with participants that had completed both baseline and follow-up measurements. In addition, a per-protocol analysis was conducted with participants that had participated at least once compared to controls.

In the second set of analyses, cases with baseline mean scores below 75 on the total PedsQL for children and total mean scores below 4.99 on the Gothenburg QOL for adults were selected. These analyses aimed to detect potential differential effects of the intervention on participants with low HRQOL at baseline. The selection was based on the proportion of baseline scores; scores below 4.99 and 75 represented the lowest third.

Pearson’s correlation was performed between children’s and parents’ mean and maximum HRQOL scores at baseline. A mean score was calculated when two parents had participated and had data on HRQOL. If only one parent participated, that score was used in the analysis. Maximum score refers to the highest parental score, that could be either parent. The significance level was set at *p* < 0.05 for all analyses.

### Ethical considerations

Eligible families received verbal and written information about the study and gave informed consent in writing before participation in the study. Parents gave written informed consent for their children. All participants were informed that participation was voluntary and that their participation in the study would not affect their ability to participate in the programme *A Healthy Generation*. The study was approved by the Regional Ethical Review Board in Stockholm (2016/447–31/2, 2016/1254–32 and 2017/2379–32) and follows the ethical principles of the Declaration of Helsinki 1964.

## Results

### Baseline characteristics

Descriptive characteristics of participating children and adults at baseline are presented in Table 1 and Table 2, respectively. At baseline there were no statistically significant differences between participating children or adults in the intervention and the control groups in terms of gender, age, parental education, country of birth, anthropometry or HRQOL baseline scores. Participating children’s mean age at baseline was 8.2 ± 0.3 years and the age range was 7.7–9.2 years. Of the children in the study, 45% were girls: 50% girls in the intervention group and 39% girls in the control group. Participating adults’ mean age at baseline was 39.5 ± 6.8 years and the age range was 25–55 years. Among adults in the study, 64% were women, 62% women in the intervention group and 67% women in the control group. Of the participating adults, 59% were born outside Sweden.

**Table 1:** Baseline characteristics of all participating children and for the children in the intervention and control group separately

Children	Total ***n*** = 67	Intervention ***n*** = 36	Control ***n*** = 31	n (I/ C)	p
	Mean (SD)	Mean (SD)	Mean (SD)		
Age (years)	8.2 (0.3)	8.2 (0.3)	8.2 (0.3)	67 (36/31)	0.47
Female (%)	45%	50%	39%	30 (18/12)	0.35
Parental low education (%)	63%	68%	59%	40 (23/17)	0.46
Participation, nr/total nr. (sd)		31/65 (18)			
Anthropometry					
Weight (kg)	29.5 (7.3)	27.9 (6.1)	31.2 (8.0)	63 (32/31)	0.07
Height (cm)	130.3 (5.9)	129.8 (6.1)	131.0 (5.9)	65 (35/30)	0.44
Waist circumference (cm)	62.2 (8.1)	60.8 (7.1)	63.9 (8.9)	67 (36/31)	0.12
Body mass index	17.2 (3.2)	16.5 (2.4)	18.0 (3.7)	62 (32/30)	0.07
HRQOL					
PedsQL total	79 (11)	81 (11)	78 (11)	64 (34/30)	0.33
PedsQL psychosocial	76 (12)	76 (12)	75 (12)	63 (33/30)	0.79
PedsQL physical	86 (12)	88 (11)	83 (13)	65 (35/30)	0.07
PedsQL emotional	69 (18)	72 (18)	66 (18)	64 (34/30)	0.20
PedsQL social	80 (17)	79 (17)	81 (17)	63 (33/30)	0.53
PedsQL school	78 (14)	78 (14)	79 (14)	63 (33/30)	0.89

**Table 2:** Baseline characteristics of all participating adults and for adults in the intervention and control group separately

Adults	Total ***n*** = 90	Intervention ***n*** = 47	Control ***n*** = 43	n (I/ C)	p
	Mean (SD)	Mean (SD)	Mean (SD)		
Age (years)	39.5 (6.8)	39.5 (6.4)	39.5 (7.2)	90 (47/43)	0.98
Female (%)	64%	62%	67%	58 (29/29)	0.57
Low education (%)	66%	72%	63%	59 (33/26)	0.41
Born outside of Sweden (%)	59%	57%	62%	53 (27/26)	0.67
Participation, nr/total nr. (sd)		25/65 (15)			
Anthropometry					
Weight (kg)	78.2 (19.0)	81.8 (21.6)	74.9 (15.8)	83 (41/42)	0.11
Height (cm)	167.4 (9.2)	168.9 (8.6)	165.9 (9.8)	90 (47/43)	0.13
Waist circumference, male (cm)	100.4 (15.9)	102.0 (18.0)	98.4 (13.2)	32 (18/14)	0.53
Waist circumference, female (cm)	91.1 (14.7)	91.7 (13.0)	90.6 (16.4)	55 (28/27)	0.73
Body mass index	27.7 (6.2)	28.5 (7.5)	27.0 (4.7)	83 (41/42)	0.30
HRQOL					
HRQOL total	5.3 (0.9)	5.3 (0.9)	5.2 (1.0)	89 (47/42)	0.59
HRQOL physical	5.3 (1.0)	5.4 (1.0)	5.3 (0.9)	89 (47/42)	0.44
HRQOL social	5.3 (1.2)	5.3 (0.9)	5.2 (1.4)	89 (47/42)	0.58
HRQOL mental	5.2 (1.1)	5.2 (1.1)	5.2 (1.2)	89 (47/42)	0.94

### Intervention effects on quality of life

**Fig. 2:**
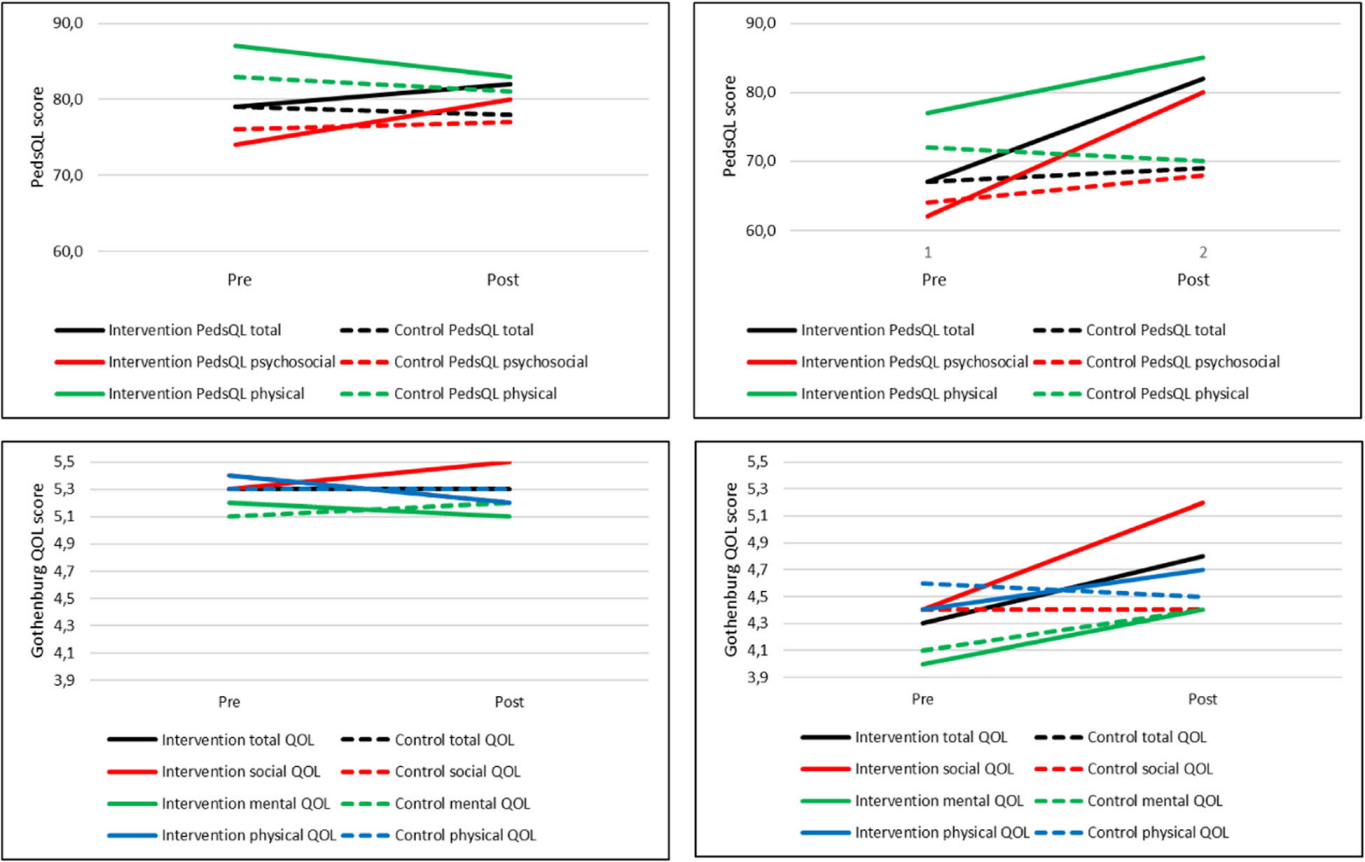
Pre- and post-intervention measure of HRQOL for children (top) and adults (bottom) in the total sample (left) and the sub-sample of participants who had low HRQOL at baseline (right)

Effects of the intervention on HRQOL are shown in Fig. 2 and presented in Table 3 for the whole sample of children and adults and in Table 4 for the subgroup of children and adults with initial low baseline scores on QOL. After adjustment for baseline values, there were no significant differences in HRQOL between intervention and control groups in the total sample of children (*n* = 56) or adults (*n* = 69). Analyses including only participants that had participated at least once (children *n* = 26 adults *n* = 34), compared to the control group did not change the results. In a subgroup of children (*n* = 20) with initial low HRQOL scores at baseline, there was a significantly higher HRQOL than the corresponding control group after the intervention in total score (13.2 adjusted mean difference, 95% CI 2.1–24.3, *p* = 0.02), psychosocial score (13.8 adjusted mean difference, 95% CI 1.4–26.2, *p* = 0.03) and physical score (16 adjusted mean difference, 95% CI 5.2–26.8, *p* = 0.01). Also, in a subgroup of adults (*n* = 21) with initial low HRQOL at baseline there was a significantly higher social HRQOL (0.9 adjusted mean difference, 95% CI 0.0–1.7, *p* = 0.04), than the control group after the intervention.

**Table 3:** Change in HRQOL and QOL from baseline to follow-up among participating children and adults

	Intervention		Mean (SD)		Control	Mean (SD)	Mean difference adjusted for baseline	
	n	Baseline	Follow-up	n	Baseline	Follow-up	(95% confidence interval)	p
Children								
PedsQL total	28	79 (10)	82 (12)	28	79 (11)	78 (13)	3.1 (−3.2–9.3)	0.33
PedsQL psychosocial	27	74 (12)	80 (15)	28	76 (12)	77 (16)	4.3 (−2.9–11.5)	0.23
PedsQL physical	29	87 (11)	83 (10)	28	83 (13)	81 (14)	1.7 (−4.6–7.9)	0.59
Adults								
HRQOL total	35	5.3 (0.8)	5.2 (0.9)	34	5.3 (0.9)	5.3 (1.0)	−0.7 (−0.4–0.3)	0.69
HRQOL physical	35	5.4 (1.0)	5.2 (1.0)	33	5.3 (0.8)	5.3 (1.0)	−0.2 (−0.5–0.2)	0.34
HRQOL social	35	5.3 (0.9)	5.5 (0.8)	33	5.4 (1.1)	5.2 (1.3)	0.3 (−0.1–0.7)	0.15
HRQOL mental	35	5.2 (1.0)	5.1 (1.3)	33	5.1 (1.1)	5.2 (1.0)	−0.2 (− 0.7–0.2)	0.30

**Table 4:** Change in HRQOL from baseline to follow-up among participating children and adults with initial low scores

	Intervention		Mean (SD)		Control	Mean (SD)	Mean difference adjusted for baseline	
	n	Baseline	Follow-up	n	Baseline	Follow-up	(95% confidence interval)	p
Children								
PedsQL total	9	67 (6)	82 (11)	11	67 (5)	69 (12)	13.2 (2.1–24.3)	0.02^a^
PedsQL psychosocial	9	62 (5)	80 (12)	11	64 (6)	68 (17)	13.8 (1.4–26.2)	0.03^a^
PedsQL physical	9	77 (12)	85 (12)	11	72 (11)	70 (9)	16.0 (5.2–26.8)	0.01^a^
Adults								
HRQOL total	10	4.3 (0.5)	4.8 (0.8)	12	4.4 (0.5)	4.4 (1.0)	0.5 (−0.3–1.2)	0.24
HRQOL physical	10	4.4 (0.9)	4.7 (0.8)	12	4.6 (0.6)	4.5 (1.0)	0.4 (−0.4–1.2)	0.32
HRQOL social	10	4.4 (1.0)	5.2 (0.7)	11	4.4 (0.7)	4.4 (1.3)	0.9 (0.0–1.7)	0.04^a^
HRQOL mental	10	4.0 (0.7)	4.4 (0.8)	12	4.1 (0.8)	4.4 (1.1)	0.0 (−1.0–1.2)	0.86

^a^Significant difference between intervention- and control groups

### Participation and drop-outs in the intervention group

There were 36 children in grade 2 and 47 adults in the intervention group. In total, 65 activity sessions were provided for each intervention group. Mean (SD) participation among those who participated at least once was 31 (±18) times (range: 4–64) among children and 25 (±15) times (range: 1–58) among adults. Participation was higher on weekdays than on weekends for both children and adults. Mean (SD) participation for children was 18 (±21) times (range 0–33) on weekdays and 13 (±13) times (range: 1–31) on weekends. Adults’ mean (SD) participation was 14 (±17) times (range: 0–31) on weekdays and 11 (±8) times (range: 0–30) on weekends. Seven participants (4 adults and 3 children) in the intervention group did not participate in the programme activities and another six children and nine adults participated fewer than 10 times. Children and adults who participated fewer than 10 times were similar to the total sample in terms of sociodemographic characteristics such as sex, BMI and parental education, as well as baseline HRQOL scores.

The level of participation in *A Healthy Generation* was significantly and positively correlated with a change in physical HRQOL scores among girls (*r* = 0.44, *p* = 0.01), but not boys (*r* = − 0.07, *p* = 0.58). A similar trend was seen among participating mothers, where the correlation between level of participation and change in physical HRQOL scores almost reached significance (*r* = 0.42, *p* = 0.05), but not for fathers. In total 8/36 children and 20/90 adults did not have complete data on HRQOL on follow-up. There was no statistically significant difference between participants without complete data versus the control group or versus participants with complete data in terms of sociodemographic characteristic or baseline HRQOL score.

### Family correlations in HRQOL

At baseline, there was a significant correlation in HRQOL total scores between children’s HRQOL and their parents’ mean HRQOL score (*r* = 0.38, *p* < 0.001), as well as between children’s HRQOL and the parent with the highest score (*r* = 0.41, *p* < 0.001). With regard to the different domains of PedsQL, there was a significant correlation between parents’ mean and max HRQOL score and their children’s mean scores in the physical (*r* = 0.34, *p* < 0.001), social (*r* = 0.30, *p* = 0.02), emotional (*r* = 0.36, *p* = 0.02) and psychosocial domains (*r* = 0.36, *p* < 0.001), but not for the school domain (*r* = 0.13, *p* = 0.30).

## Discussion

A programme aiming at supporting an active and healthy lifestyle delivered in socioeconomically disadvantaged areas in Sweden revealed no significant differences in HRQOL in the total study-sample. However, in children and adults with low HRQOL a significantly positive increased HRQOL were noted. The study also demonstrated a statistically significant positive correlation within families between the parents’ HRQOL and their child’s HRQOL at baseline. Furthermore, within the intervention group, there was a significant positive relationship between level of participation in *A Healthy Generation* and changes in the physical domain of HRQOL among girls but not boys.

The programme *A Healthy Generation* is unique in that it is both directed to the whole family and involves broad collaboration at the municipality level, including health coordinators, local enterprises and sport associations. To our knowledge, no previous studies have reported results on how such a family-focused health promotion programme affects HRQOL. The focus on disadvantaged areas is also novel. Previous studies have either been school-based [41, 42] or involved a community approach, but with older participants [30]. Our findings on the correlation of HRQOL within families at baseline stress the importance of involving the entire family in health promotion interventions. Children’s health has been reported to affect and to be affected by their families and their life situations [43, 44], however; research to identify relevant family correlations of these aspects is sparse. Studies on correlations of HRQOL between family members have mainly focused on specific samples of children, such as children with psychiatric symptoms [45]. The instruments used in this study contain no questions related to family relations. Given that the intervention was directed to the whole family and had a specific parental component, it would have been interesting to use an instrument measuring family relations such as the KIDSCREEN 27 questionnaire [3].

The significant results, even though it concerned a small subgroup of children and adults with initial low HRQOL scores at baseline can be relevant to consider in relation to inequalities in health. Improving health among those who need it most is important from an equality perspective. Differences in mental health with regard to socioeconomic factors are seen from an early age. Research has highlighted an association between mental health problems and socioeconomic factors such as low parental education and income, in both children and their parents [9, 10, 46]. It is also well known that both children with chronic conditions and their parents more commonly have impaired HRQOL [43, 44]. The level of HRQOL reported by the sub-sample of children with initial low HRQOL scores at baseline in this study was similar to that observed in paediatric patients, such as those with cancer and rheumatological conditions [43]. Self-reported low HRQOL has considerable implications for health. In paediatric populations, assessment of HRQOL is particularly important in order to identify hidden or unexpected health problems, subgroups with poor health, and to identify health inequalities [47]. This has prompted the Public Health Agency of Sweden to stress the importance of both monitoring mental health in vulnerable groups, and finding strategies to promote mental health [37].

Our results also show a significant relationship between level of participation in *A Healthy Generation* and the physical domain of HRQOL among girls in the intervention group. These results are interesting, especially from a gender perspective. Recent research has highlighted both that girls are less physically active than boys, and that girls report more psychosocial health complaints than boys [5–8, 24]. There is an urgent need to find strategies to improve HRQOL and prevent mental health problems, particularly among girls. The correlation between participation in the intervention and changes in physical HRQOL among girls is therefore important to further explore.

### Strengths and limitations

The strengths of this study include the controlled study design in a real life setting with an intervention directed to socioeconomically disadvantaged families. The control group was carefully selected to match the intervention group on socioeconomic factors and there were no baseline differences between intervention and control groups. Moreover, HRQOL was measured with two validated instruments: PedsQL and Gothenburg QOL.

With regard to limitations, it was not possible to randomise the groups, since the schools where the programme was delivered were strategically selected by the municipality. To randomise individuals within schools, i.e. to offer the programme to some children but not to others, were not performed for ethical reasons. Furthermore, the number of participants included in this study was limited by how many participants were available at the selected study site. Other intervention studies investigating HRQOL in children have had more participants [30, 48]. It may be a challenge to improve HRQOL in a normal healthy population. At baseline, there was a ceiling effect in PedsQL, where participants – especially children – scored high on all domains. Compared with other instruments measuring HRQOL in children and adolescents, PedsQL has fewer items covering positive aspects of the included domains [49]. An instrument with more positively worded items might have detected more changes of HRQOL in a positive direction.

## Conclusions

The study showed no significant effects on HRQOL in the whole study sample. The findings suggest that participation in the pilot intervention *A Healthy Generation* may increase HRQOL for children and adults with low HRQOL. The study was conducted at one study site within the programme *A Healthy Generation*, which is currently being delivered in several in Sweden. The limited sample size means that the findings of this study need to be interpreted with caution. Further studies, preferably with a larger study population, are needed to explore the effect of health promoting interventions directed to families on HRQOL in disadvantaged areas. This knowledge can contribute to the development of health promoting interventions in such areas.

## Data Availability

The datasets used and analyzed during the current study are available from the corresponding author on reasonable request.

## Abbreviations

HRQOL: Health Related Quality of Life
PedsQL: Pediatric Quality of Life Inventory
QOL: Quality of Life
